# Supplementary material for: Calibration-free NGS quantitation of mutations below 0.01% VAF
Source: Nat Commun. 2021 Oct 21;12:6123. doi: 10.1038/s41467-021-26308-6 (PMC8531361; doi:10.1038/s41467-021-26308-6)
Supplement: Supplementary file 1 — Supplementary Information [file 41467_2021_26308_MOESM1_ESM.pdf]

Supplementary Information for

Calibration-free NGS Quantitation of Mutations below 0.01% VAF

Peng Dai[1,2,3], Lucia Ruojia Wu[1,2], Sherry Xi Chen[1,3], Michael Xiangjiang Wang[1], Lauren Yuxuan Cheng[1], Jinny Xuemeng Zhang[4], Pengying Hao[4], Weijie Yao[4], Jabra Zarka[5], Ghayas C. Issa[5], Lawrence Kwong[6], David Yu Zhang[1,3,7*]

[1] Department of Bioengineering, Rice University, Houston, TX, USA.

[2]These authors contributed equally: Peng Dai, Lucia Ruojia Wu

[3]Present affiliation: NuProbe USA, Houston, TX, USA.

[4] NuProbe USA, Houston, TX, USA.

[5]Department of Leukemia, The University of Texas MD Anderson Cancer Center, Houston, TX, USA.

[6]Department of Translational Molecular Pathology, The University of Texas MD Anderson Cancer Center, Houston, TX, USA.

[7] Systems, Synthetic, and Physical Biology, Rice University, Houston, TX, USA.

*e-mail: genomic.dave@gmail.com

Supplementary Note 1. Single-plex QBDA

To demonstrate the QBDA, an 18-nt region in tuberculosis rpoB (Rv0667) gene (NC_000962.2_761099:761116) were chosen as the enrichment region. Nine different drug resistance mutations in the enrichment region were picked, including single-base substitution, insertion and deletion. Gene block (gBlock) from Integrated DNA Technologies was synthesized for each mutation, with mutated enrichment region and wild type (WT) sequence flanking around (Supplementary Figure 1). Tuberculosis laboratory strain H37Rv (ATCC 27294) served as wild type. Nine synthetic gBlocks were quantified and mixed with H37Rv strain for a spike-in reference DNA sample with approximately 1% VAF for each mutation. The gBlocks were first diluted to 10 ng/uL in IDTE following instruction from IDT, then further diluted with Tris EDTA solution with 0.1% tween and 100 ng/uL carrier RNA (Qiagen catalog # 1068337) before mixing with WT DNA. All mutations were simultaneously enriched and accurately quantified (Fig 1b).

a

Mutation Entry	Mutation position	Gene name	Nucleic acid change	Amino acid change
1	NC_000962.2_761099	rpoB	C>G	S512R
2	NC_000962.2_761100	rpoB	C>A	Q513K
3	NC_000962.2_761101	rpoB	A>C	Q513P
4	NC_000962.2_761103	rpoB	InsTTC	F514_Ins
5	NC_000962.2_761108	rpoB	G>T	M515I
6	NC_000962.2_761109	rpoB	G>T	D516Y
7	NC_000962.2_761110	rpoB	A>G	D516G
8	NC_000962.2_761110	rpoB	A>T	D516V
9	NC_000962.2_761112:761114	rpoB	DelCAG	Q517_Del

b

```
       WT: CCAATTCATGGACCAGAA
    S512R: GCAATTCATGGACCAGAA
    Q513K: CAAATTCATGGACCAGAA
    Q513P: CCCATTCATGGACCAGAA
 F514_Ins: CCAATTCTTCATGGACCAGAA
    M515I: CCAATTCATTGACCAGAA
    D516Y: CCAATTCATGTACCAGAA
    D516G: CCAATTCATGGGCCAGAA
    D516V: CCAATTCATGGTCCAGAA
 Q517_Del: CCAATTCATGGAC---AA
```

Supplementary Figure 1. WT and 9 spike-in mutations sequence in enrichment region. The enrichment region is NC_000962.2_761099:761116 in tuberculosis rpoB (Rv0667) gene.

Supplementary Table 1. Primer sequences for tuberculosis QBDA

BDA_fp	GGCACCAGCCAGCTGAG
Sfp	GGATATTCCTTTCTACTCTTTGACATCATCTATCACTTC GGCACCAGCCAGC
Blocker	CAGCTGAGCCAATTCATGGACCAGAA/iSpC3//iSpC3/AT
Srp	AGACGTGTGCTCTTCCGATCTATCAHHHHHHHHHHHHH HHCCGACAGTCGGCGCTT
Adp_fp	ACACGACGCTCTTCCGATCTGGCACCAGCCAGCTGAG
Universal Forward Primer (Ufp)	CCTATGGTAGTTAAATGTACATTGGATATTCCTTTCTAC TCTTTGACATCATCT
Universal Reverse Primer (Urp)	GACTGGAGTTCA**GACGTGTGCTCTTCCGATCT**

Supplementary Note 2. Multiplex QBDA SNP panel

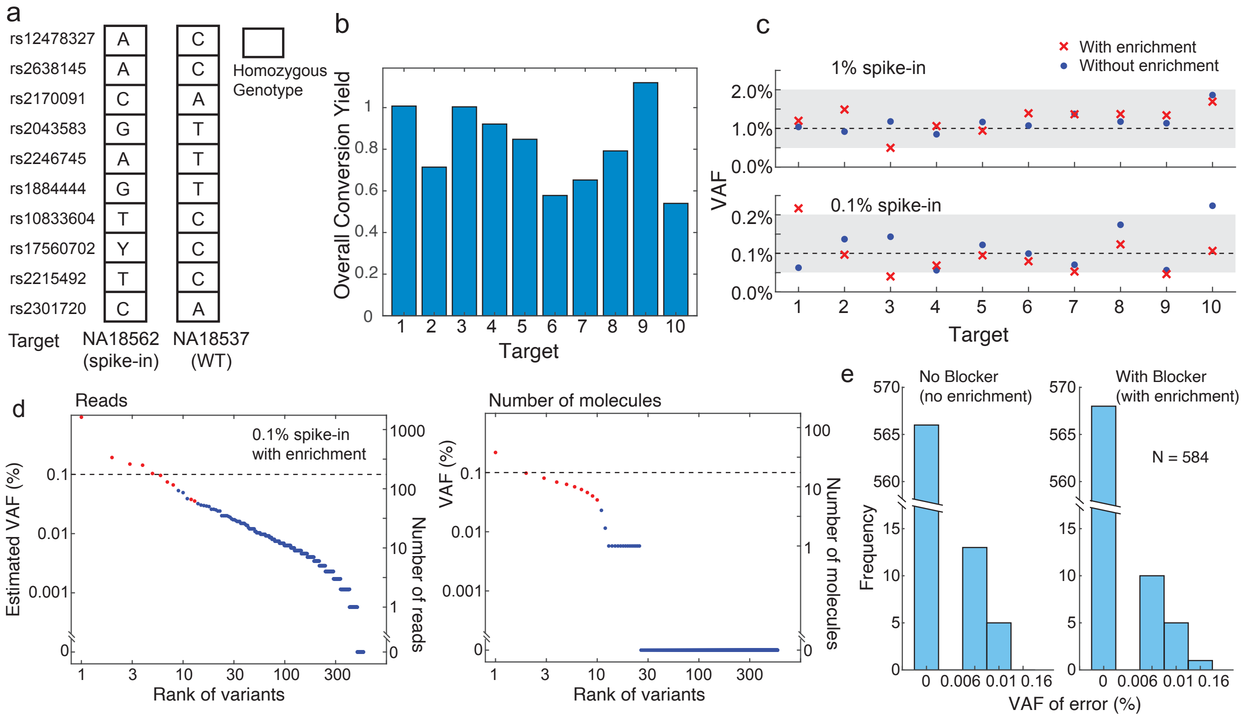

Supplementary Figure 2. QBDA quantitation validation in 10-plex SNP panel. a. Information for SNPs covered in 10-plex SNP panel. b. Conversion yield for each amplicon calculated from no enrichment library. c. Quantitation accuracy for 1% spike-in and 0.1% spike-in. Libraries with and without enrichment were compared. d. UMI significantly eliminated errors as demonstrated by the sorted variants observed in reads and UMI. The ten red dots are expected mutations from NA18562 spike-in, and all blue dots are false positive errors. e. Variant enrichment does not lead to higher error rate comparing to no enrichment.

Supplementary Table 2. Primer sequences for QBDA SNP panel
Provided in Supplementary Data 1

Supplementary Note 3. Multiplex QBDA Leukemia panel

Supplementary Table 3. Mutations covered in QBDA Leukemia panel

Plex number	Gene	Amino Acid Change	Expect nucleic acid change in enrichment region	Expected VAF in mixed reference standard
1	ABL1	T315I	1C>T	2.5%
2	ASXL1	W796C	4G>T	2.5%
3	BCOR	Q1174fs*8	2InsT	35%
4	CBL	S403F	6C>T	2.5%
5	DNMT3A	R882C	2G>A	2.5%
6	EZH2	R418Q	2C>T	2.5%
7	FLT3	D835Y	14C>A	2.5%
8	GATA1	Q119*	4C>T	5%
9	IDH1	R132C	4G>A	2.5%
10	IDH2	R172K	2C>T	2.5%
11	JAK2	F537-K539>L	5DelTCACAA	2.5%
12	JAK2	V617F	2G>T	2.5%
13	KRAS	G13D	1C>T	20%
14	NPM1	W288fs*12	2InsCAGA	2.5%
15	NRAS	Q61L	1A>T	5%
16	RUNX1	M267I	2C>T	17.5%
17	SF3B1	G740E	9C>T	2.5%
18	TET2	R1261H	1G>A	2.5%
19	TP53	S241F	9G>A	2.5%
20	IDH2	R140	1C>T	5%
21	KIT	D816	3A>T	5%
22	ETV6	R369Q	12G>A	5%

Supplementary Table 4. Primer sequences for QBDA Leukemia panel
Provided in Supplementary Data 2

3.1 Spike-in positive sample

Mutations 1-19 are present in Myeloid DNA Reference Standard (Horizon Discovery Ltd., Catalog ID: HD829), with VAF between 5%-70% for different mutations. Gblocks from Integrated DNA Technologies were synthesized for mutations 20-22. The diluted gblocks were mixed with Myeloid DNA Reference Standard and NA18562 genomic DNA to yield mixed reference standard for an expected VAF of 2.5%-35% for mutations 1-19, and approximately 5% for mutations 20-22.

The mixed reference standard was diluted with human genomic DNA from healthy people to yield lower VAF samples. For example, 4 ng mixed reference standard was mixed with

1000 ng healthy gDNA to yield a DNA sample with expected mutation VAF between 0.01% and 0.14%.

3.2 Supplement analytical performance: LoD for 1 µg DNA input

The defined LoD threshold is not the same for all types of mutations. We use 0.003% for most types of single-base substitutions, and 0.006% for C>T/G>A and T>C/A>G types of mutations.

This was based on the observations from 10 different healthy PBMC DNA samples (Supplementary Fig. 3). The most commonly observed mutations are C>T/G>A and T>C/A>G, even after applying the count filter to remove mutation calls with <6 UMI family count. This result indicates that C>T/G>A and T>C/A>G mutations are likely prevalent in polymerase error, DNA damage and/or clonal hematopoiesis-induced variants.

Polymerase error rate varies for different mutations[1]. Based on our past experience, PCR and NGS are prone to homopolymer indel errors (i.e. length change of ≥4 nt homopolymers), so we used 0.006% LoD threshold for homopolymer indels. We set LoD at 0.0006% for long indels and complex mutations which have very low chance of PCR or NGS error.

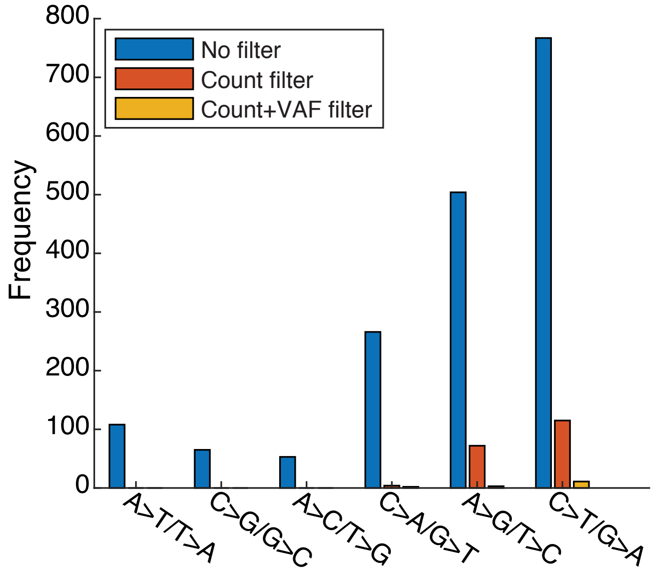

Supplementary Figure 3: Single-base substitutions classified by type. 10 PBMC gDNA samples from different healthy donors were analyzed using QBDA AML panel (500 ng per library), and the frequency of different types of single-base substitutions are plotted as bar graph. After count filter to remove mutations with < 6 UMI count, mutations in these samples indicate potential DNA damage and/or clonal hematopoiesis.

3.3 Minimum required depth analysis using *in silico* down-sampling

In order to analyze the minimum required sequencing depth for 0.01% VAF, we sequenced the positive sample in Fig. 2b using QBDA with excess sequencing depth, and performed *in silico* random down-sampling of NGS reads so that lower sequencing depth can be analyzed. The experimental sequencing depth was 350,000X (7.7 M reads). 20 simulations were performed to randomly sample 1.0 M reads from the original library to generate 45,000X depth libraries. None of the observed UMI counts dropped below 6 in the 20 simulations (i.e. no drop-out); the median UMI counts of 45,000X depth were between 38.6% and 100.0% of the UMI counts of 350,000X depth; only 1 mutation was below 50% (Fig. 2d). Therefore, we conclude that 45,000X depth has similar performance to 350,000X depth, and thus can be used for 0.01% VAF detection. A similar analysis for 23,000X depth (0.5 M reads) also had no drop-out in 20 simulations, with median UMI counts between 15.8% and 96.9% of 350,000X depth (Supplementary Fig. 4). Though 23,000X depth showed <50% conversion yield for 4 amplicons, no drop-out was observed in 20 independent simulations, with median UMI counts between 15.8% and 96.9% of 350,000X depth. 23,000X depth is still acceptable for detection of 0.01% VAF, but will recommend 45,000X depth for accurate quantitation.

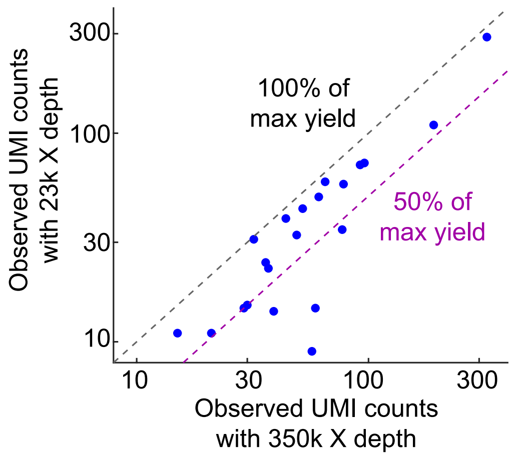

Supplementary Figure 4. *In silico* down-sampling to 23,000X sequencing depth. The 1X VAF positive sample (500 ng input) was sequenced with 350,000X depth (7.7 M reads). We performed a virtual experiment of 23,000X depth sequencing by random sampling 0.5 M reads from the original library; the median observed UMI counts of 20 independent simulations were plotted against observed UMI counts in the original library. All mutations can be observed at 23,000X depth.

3.4 **Clinical MRD analysis**

Supplementary Table 5. Population characteristics, molecular and clinical information of the leukemia patients
Provided in Supplementary Data 3

Supplementary Table 6. QBDA test results for the 10 paired samples from 5 leukemia patients
Provided in Supplementary Data 4

Supplementary Note 4. Multiplex QBDA pan-cancer panel

A pan-cancer panel (VarMap™ Pan-Cancer NGS Panel) is developed by NuProbe Inc. based on QBDA technology.

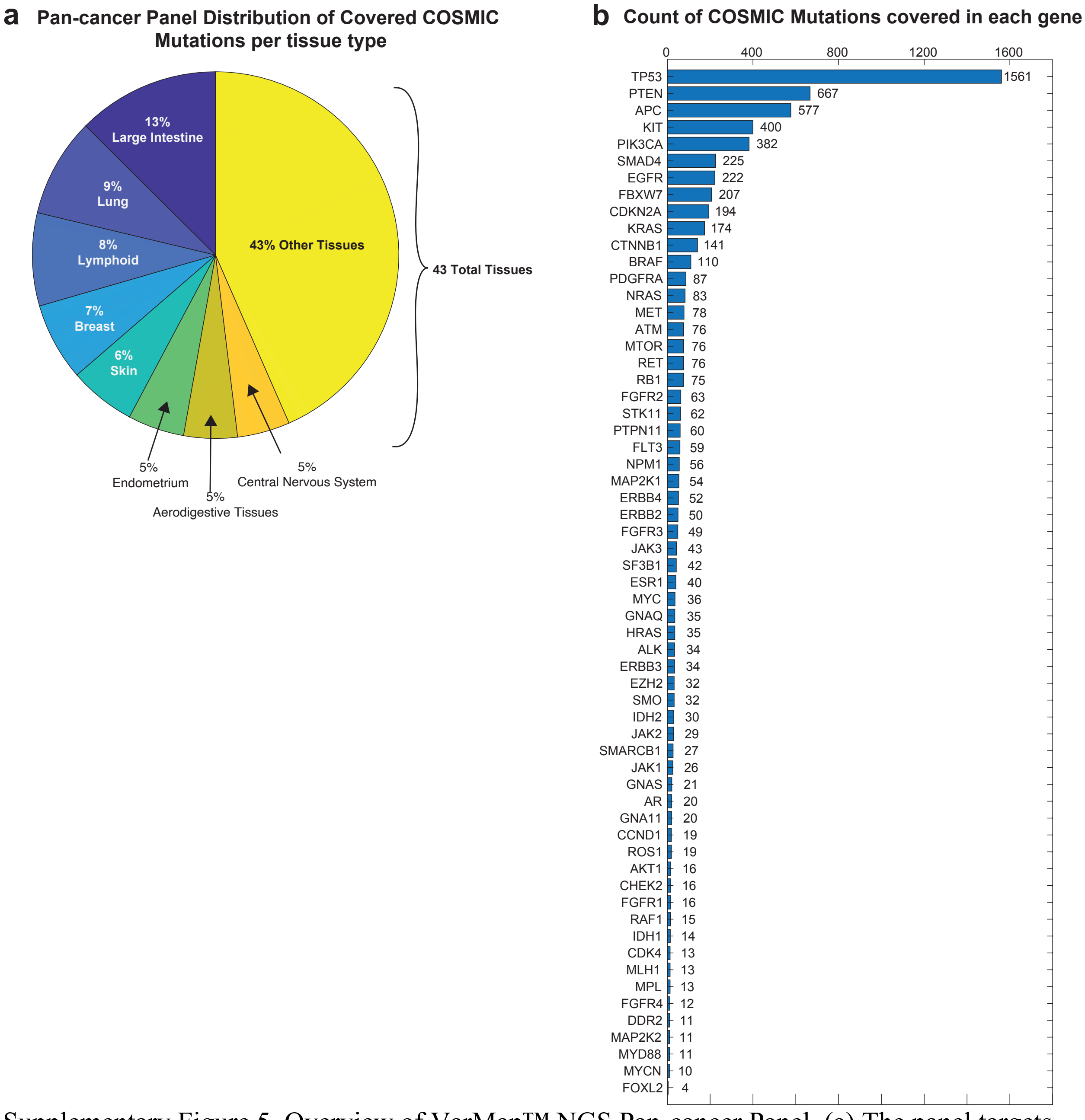

Supplementary Figure 5. Overview of VarMap™ NGS Pan-cancer Panel. (a) The panel targets somatic mutations across 43 different tissues, focusing on the most common cancer tissues in US. (b) The panel targets a total of 7276 COSMIC variants across 61 genes.

Supplementary Table 7. List of variants included in the positive control.

Gene	COSMIC ID	Mutation CDS	Mutation AA	Mutation Description
AKT1	COSM33765	c.49G>A	p.E17K	Substitution - Missense
EGFR	COSM12382	c.2239_2248TTAAGAGAAG>C	p.L747_A750>P	Complex - deletion
ERBB2	COSM436498	c.2033G>A	p.R678Q	Substitution - Missense
ESR1	COSM4745827	c.908A>G	p.K303R	Substitution - Missense
ESR1	COSM3829320	c.1138G>C	p.E380Q	Substitution - Missense
GNAQ	COSM404628	c.286A>T	p.T96S	Substitution - Missense
GNAQ	COSM6304069	c.175A>C	p.M59L	Substitution - Missense
KIT	COSM1304	c.1924A>G	p.K642E	Substitution - Missense
MAP2K1	COSM235614	c.370C>T	p.P124S	Substitution - Missense
MTOR	COSM4187184	c.7255G>A	p.E2419K	Substitution - Missense
MTOR	COSM462601	c.5930C>A	p.T1977K	Substitution - Missense
PDGFRA	COSM736	c.2525A>T	p.D842V	Substitution - Missense
PIK3CA	COSM773	c.3129G>T	p.M1043I	Substitution - Missense
RAF1	COSM181063	c.770C>T	p.S257L	Substitution - Missense
PTEN	COSM5219	c.388C>G	p.R130G	Substitution - Missense
PTEN	COSM5154	c.697C>T	p.R233*	Substitution - Nonsense
TP53	COSM10660	c.818G>A	p.R273H	Substitution - Missense
FBXW7	COSM22975	c.1513C>T	p.R505C	Substitution - Missense
FBXW7	COSM22973	c.1177C>T	p.R393*	Substitution - Nonsense
PTPN11	COSM14271	c.1508G>T	p.G503V	Substitution - Missense

Supplementary Table 8. Pan cancer panel test results for the 16 clinical samples.
Provided in Supplementary Data 5

Supplementary Note 5. Multiplex QBDA Melanoma panel

We built a melanoma panel covering 22 hotspot regions in 8 genes (Supplementary Fig. 6a and Supplementary Table 9-10). Low input DNA (6-20 ng) is used as input for detecting > 0.1% VAF. 16 FFPE and 7 FF tissue samples from melanoma patients were tested (Fig. 4d, Supplementary Table 11). Co-existence of *BRAF* V600E and low frequency *NRAS* Q61K mutations in FFPE5 sample was observed. Although *BRAF* and *NRAS* mutations are usually mutually exclusive in melanoma patients, *BRAF/NRAS* dual mutation may derive from two subclonal populations. In addition, there were recent reports in which *BRAF* and *NRAS* co-mutations were observed in the same cell after treated with a *BRAF* inhibitor[2]. As the patient for FFPE5 was treated with *BRAF* inhibitor, the co-existence of low frequency *NRAS* indicated potential resistance mechanism related to *NRAS*. The quantitation accuracy was validated with gblock spike-in sample (Supplementary Fig. 6b) and confirmed by ddPCR in clinical DNA samples with BRAF/NRAS mutations (Supplementary Fig. 7, Supplementary Table 12). One healthy donor PBMC gDNA sample and three FFPE samples and without *BRAF/NRAS* mutation by QBDA were also tested by ddPCR, confirming no false negative were made (Supplementary Fig. 8, Supplementary Table 12).

QBDA can tolerate and normalize copy number change for accurate VAF quantitation. *BRAF* gene in melanoma FFPE12 sample underwent both copy number variation (CNV) and mutation. As demonstrated in Methods Section, the total number of UMI family count for one locus needs to be adjusted by the copy number in genome if CNV occurs. VAF for *BRAF* V600K mutation was consistent with ddPCR after normalizing the copy number of *BRAF* gene (Supplementary Fig. 7). Comparing to normal NGS without UMI and variant enrichment, QBDA reduced both false positive and false negative variant calls in the abovementioned 23 clinical samples (Fig. 4e).

To suppress the amplification of a pseudogene covered by amplicon 13 (in PIK3CA), a reverse blocker is added. The reverse blocker sequence is included in the Supplementary Table 10.

QBDA quantitation results are compared to normal NGS without UMI using the melanoma clinical samples. 10 ng to 50 ng of genomic DNA extracted from FFPE samples were used as input in multiplex PCR using the same set of primers as the melanoma 15-plex BDA panel at 50 nM each. Blockers were not included in the reaction. The following thermocycling protocol was used: 98˚C- 30s; (98˚C- 20s, 63˚C- 2 min, 72˚C- 2 min)x 15; 72˚C- 5 min. The amplicons were purified by column purification. NEBNext ultra II DNA library prep kit for Illumina (NEB # E7645S) was used for library preparation following the kit protocol. NEBNext Multiplex Oligos for Illumina (Dual Index Primers Set 1) (NEB #E7600S) were used for index PCR. Sequencing was done on Illumina MiSeq using the V2 kit. Each sample was sequenced to at least 10,000x coverage.

Supplementary Table 9. Coverage of Melanoma QBDA panel

Target	Gene	Enrichment Region (GRCh38.p12)	gBlock spike-in & COSMIC ID	
1	MAP2K1	Chr15: 66435114-66435129	K57E	COSM5369532
2		Chr15: 66436814-66436830	C121S	COSM555601
3		Chr15: 66481788-66481804	E203K	COSM232755
4	MAP2K2	Chr19: 4117540-4117558	F57V	COSM3534171
5		Chr19: 4110573-4110588	C125S	COSM5855815
6		Chr19: 4101089-4101106	E207K	COSM5574290
7	AKT1	Chr14: 104776700-104776714	Q79K	COSM159008
8	AKT3	Chr1: 243695699-243695726	E17K	COSM224779
9	NRAS	Chr1: 114716123-114716137	G12S	COSM563
10		Chr1: 114713894-114713912	Q61K	COSM580
11	KRAS	Chr12: 25245346-25245358	G12S	COSM517
12		Chr12: 25227328-25227346	Q61K	COSM87298
13	PIK3CA	Chr3: 179218291-179218309	E542K	COSM760
14		Chr3: 179234293-179234307	H1047Y	COSM774
15	BRAF	Chr7: 140753333-140753353	V600E	COSM476

Supplementary Table 10. Primer sequences in QBDA Melanoma panel
Provided in Supplementary Data 6

Supplementary Table 11. QBDA test results for the clinical samples from melanoma patients
Provided in Supplementary Data 7

a

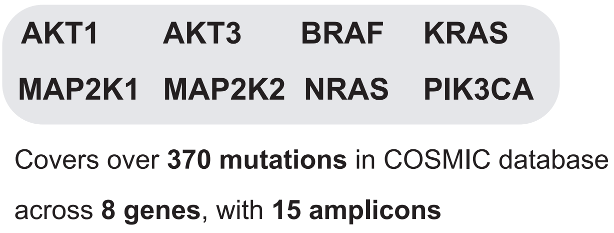

b

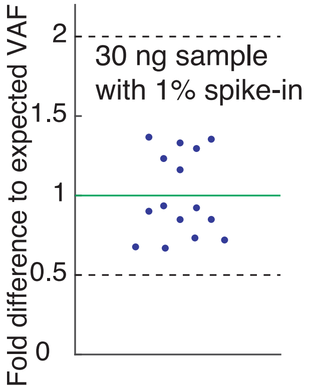

Supplementary Figure 6. QBDA Melanoma panel and clinical samples test. a. Melanoma panel coverage.

b. Quantitation accuracy validation using synthetic gBlock spike-in sample with approximately 1% VAF. VAF from QBDA is compared to the expected VAF, which is inferred by UMI-based NGS without mutation enrichment. We observed that VAF for all amplicons are about 1.57-fold higher than the expected VAF, indicating there might be systematic error in input DNA quantitation. To eliminate this systematic bias, all the QBDA VAF in melanoma panel were normalized by a factor of 1.57

Supplementary Table 12. ddPCR validation summary

	Sample ID	Mutation in Genome	Amino Acid Change	QBDA VAF%	VAF% by ddPCR	
					NRAS Q61K	BRAF V600
Positive sample (with mutation at NRAS and/or BRAF)	FFPE5	Chr1: 114713909 C>A	NRAS (c.181C>A; p.Q61K)	**0.27**	**0.02**	
		Chr7: 140753336 A>T	BRAF(c.1799T>A;p.V600E)	**35.4**		**29.48**
	FFPE20	Chr1: 114713909 C>A	NRAS (c.181C>A; p.Q61K)	**16.7**	**31.00**	
	FFPE12	Chr7: 140753336_140753337delinsTT	BRAF(c.1798_1799delinsAA;p.V600K)	**64.3***		**73.53**
Negative sample	FFPE21	No mutation in BRAF and NRAS called			**0.00**	**0.00**
	FFPE24				**0.00**	**0.00**
	FFPE10				**0.00**	**0.00**
	PBMC gDNA from healthy donor				**0.00**	

*BRAF Copy number normalized. See Supplementary Figure 7 for details of CNV confirmation and normalization.

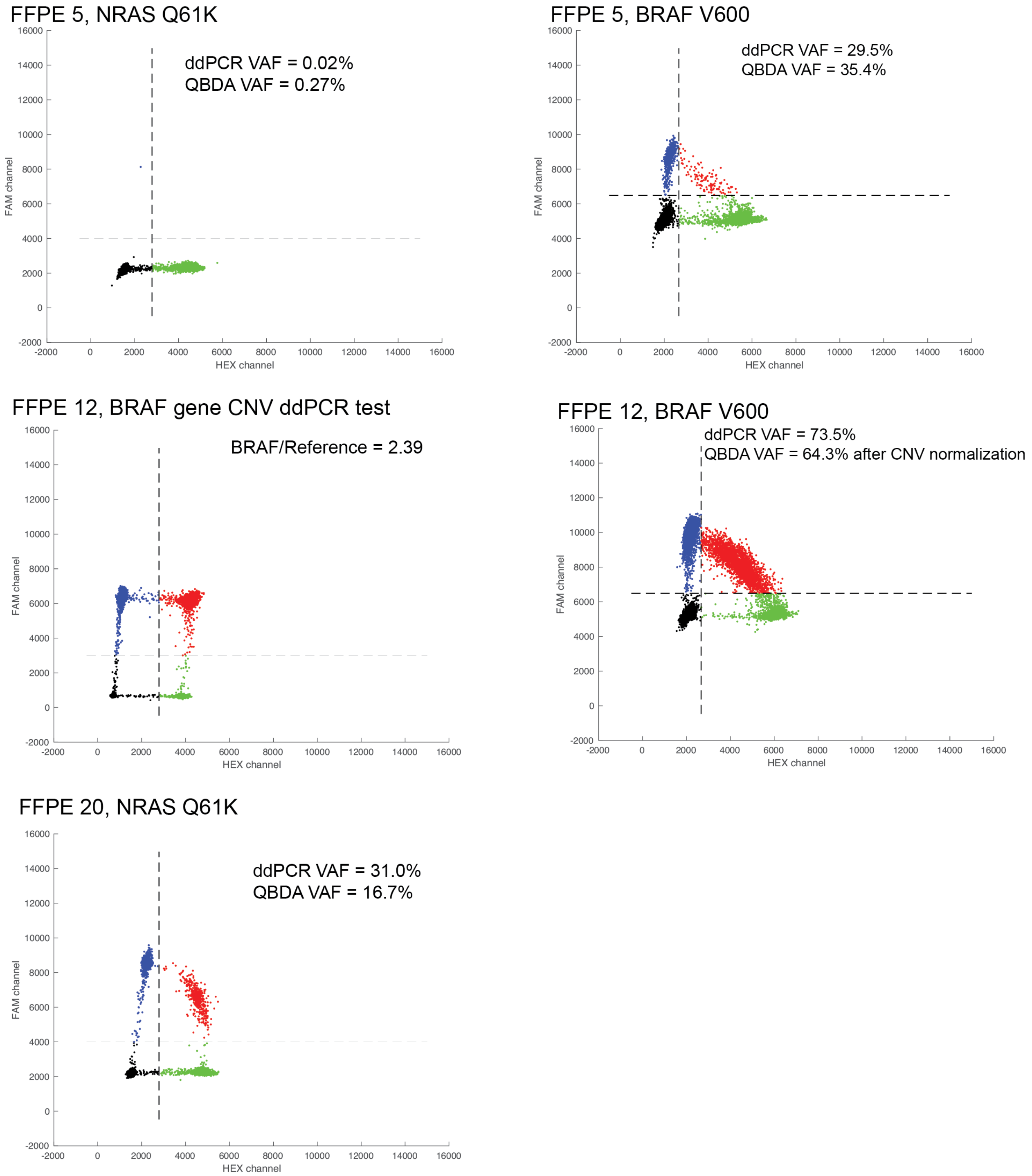

Supplementary Figure 7. ddPCR validation of observed mutations in BRAF and NRAS. The initial nominal VAF for BRAF V600K mutation in one FFPE sample (sample ID FFPE 12) is over 100%, so we suspect the copy number for BRAF gene is amplified. BRAF CNV is confirmed by BRAF CNV FAM assay using EIF2C1 HEX assay as reference. The total number of UMI family count for BRAF ($M_t = 2 * w_{input} * c_{genome} * \chi * N$) is normalized using N =

2.39. After normalization, VAF from QBDA (64.3%) is consistent with mutation VAF from ddPCR assay (73.5%).

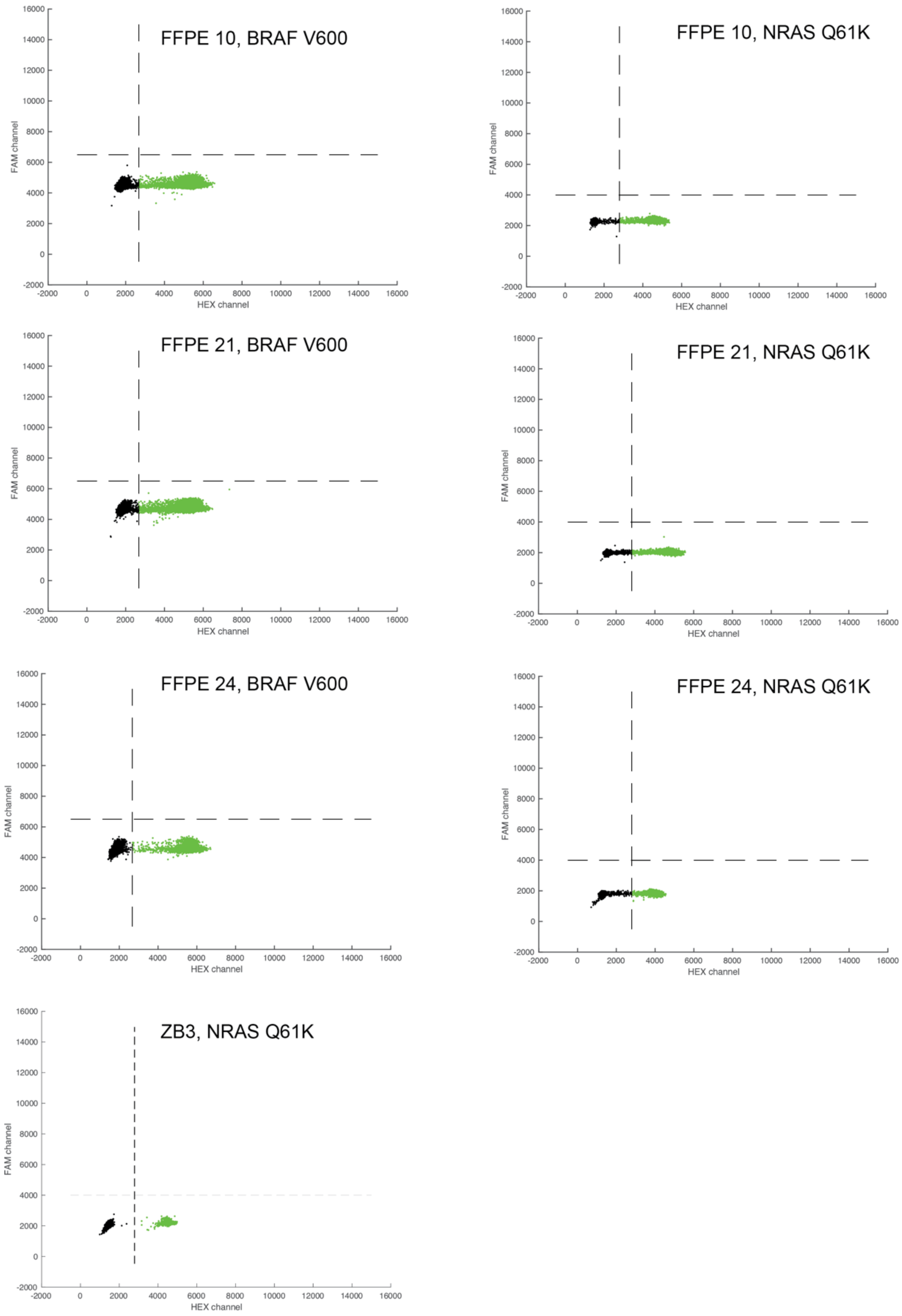

Supplementary Figure 8. ddPCR validation of QBDA negative samples. One healthy donor PBMC gDNA sample and three FFPE samples and without BRAF/NRAS mutation by QBDA were tested by ddPCR, confirming no false negative were made.

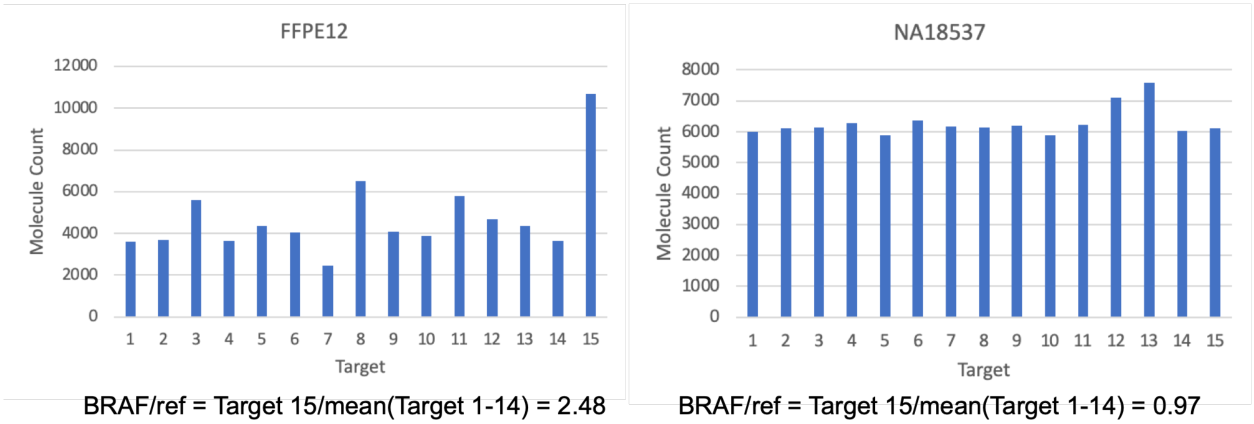

BRAF/ref = Target 15/mean(Target 1-14) = 2.48

BRAF/ref = Target 15/mean(Target 1-14) = 0.97

Supplementary Figure 9. BRAF ploidy calculation using QBDA Melanoma panel without blocker. Target 15 is an amplicon in BRAF, so BRAF copy number is calculated as the ratio between molecule count of target 15 and the mean molecule count of target 1-14. The ratio of BRAF/Ref (2.48) calculated from QBDA in FFPE 12 is consistent with ddPCR result (2.39). In a normal genomic DNA (NA18537) the ratio of BRAF/ref is close to 1.

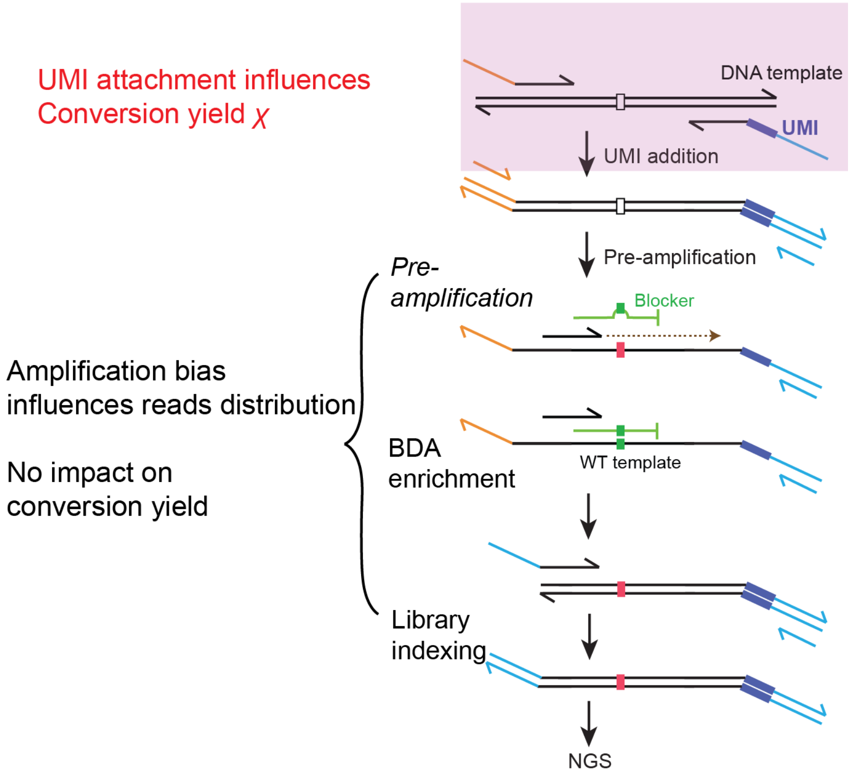

Supplementary Figure 10. Influence on conversion yield and amplification bias of each step in QBDA.

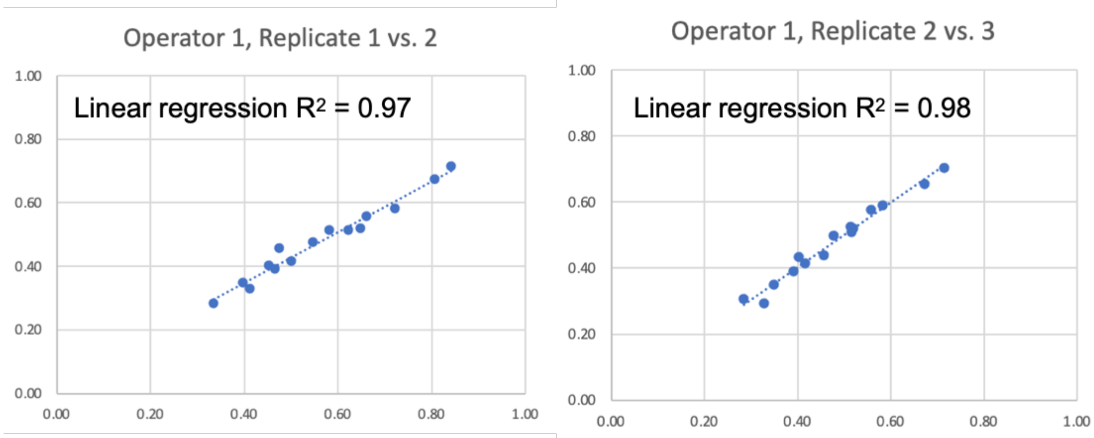

Supplementary Figure 11. Intra-operator conversion yield reproducibility of QBDA Melanoma panel in triplicate experiments.

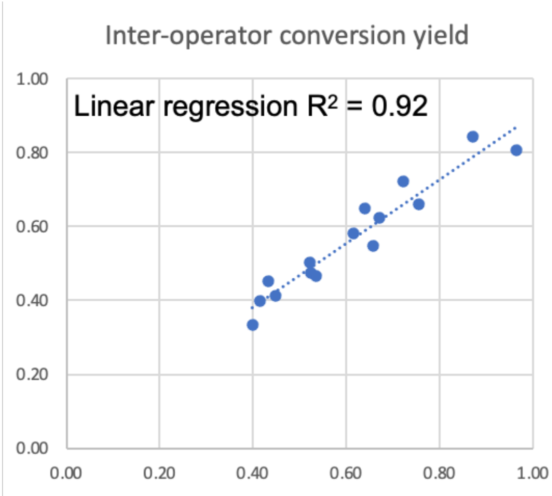

Supplementary Figure 12. QBDA Melanoma panel conversion yield is generally consistent between two different operators.

Reference:

1. Potapov, V. & Ong, J. L. Examining sources of error in PCR by single-molecule sequencing. *PLoS One* **12**, 1–19 (2017).
2. Raaijmakers, M. I. G. *et al.* Co-existence of BRAF and NRAS driver mutations in the same melanoma cells results in heterogeneity of targeted therapy resistance. *Oncotarget* **7**, 77163–77174 (2016).
