# Supplementary material for: Calibration-free NGS quantitation of mutations below 0.01% VAF
Source: Nat Commun. 2021 Oct 21;12:6123. doi: 10.1038/s41467-021-26308-6 (PMC8531361; doi:10.1038/s41467-021-26308-6)
Supplement: Supplementary file 10 — Description of Additional Supplementary Files [file 41467_2021_26308_MOESM10_ESM.pdf]

File name: Supplementary Data 1
Description: Primer sequences for QBDA SNP panel

File name: Supplementary Data 2
Description: Primer sequences for QBDA Leukemia panel

File name: Supplementary Data 3
Description: Population characteristics, molecular and clinical information of the leukemia patients

File name: Supplementary Data 4
Description: QBDA test results for the 10 paired samples from 5 leukemia patients

File name: Supplementary Data 5
Description: Pan cancer panel test results for the 16 clinical samples.

File name: Supplementary Data 6
Description: Primer sequences in QBDA Melanoma panel

File name: Supplementary Data 7
Description: QBDA test results for the clinical samples from melanoma patients

File name: Supplementary Data 8
Description: Coverage by genes and codons tested for adequate amplicons in conventional NGS panel used for AML clinical samples
